# Antiemetic prophylaxis with fosaprepitant and granisetron in pediatric patients undergoing allogeneic hematopoietic stem cell transplantation

**DOI:** 10.1007/s00432-020-03143-8

**Published:** 2020-02-13

**Authors:** Karin Melanie Cabanillas Stanchi, Julia Vek, Patrick Schlegel, Joachim Vincent Rupprecht, Tim Flaadt, Simone Weber, Sebastian Michaelis, Peter Lang, Rupert Handgretinger, Michaela Döring

**Affiliations:** 1 Department of General Pediatrics, Hematology/Oncology, University Children‘s Hospital Tübingen, Hoppe-Seyler-Str. 1, 72076 Tübingen, Germany; 2 Department of Hematology and Oncology, Olgahospital Stuttgart, Kriegsbergstrasse 60, 70174 Stuttgart, Germany

**Keywords:** Fosaprepitant, Granisetron, Pediatric, Antiemetic prophylaxis, Chemotherapy-induced nausea and vomiting, Hematopoietic stem cell transplantation

## Abstract

**Background:**

Chemotherapy-induced nausea and vomiting (CINV) is a severe and distressing complication during allogeneic hematopoietic stem cell transplantation (alloHSCT). The antiemetic fosaprepitant has shown favorable results in pediatric and adult patients receiving chemotherapy. Data on fosaprepitant in children and adolescents undergoing alloHSCT are missing.

**Methods:**

In this non-interventional observation study, 120 children and adolescents with a median age of 11.8 years undergoing alloHSCT after a moderately or highly emetogenic conditioning (MEC or HEC) were analyzed. They received an antiemetic prophylaxis with granisetron (2 × 40 µg/kg d^−1^) with or without fosaprepitant (4 mg/kg; single dose, max. 1 × 150 mg/kg BW), and were analyzed in the control (CG; *n* = 60) or fosaprepitant group (FG; *n* = 60). The efficacy and safety of the two antiemetic prophylaxis regimens were analyzed and compared with respect to the acute (0–24 h) and the delayed (> 24–120 h) CINV phase and > 120–240 h after MEC or HEC administration.

**Results:**

During MEC, significantly more patients in the CG experienced vomiting during the first 0–24 h (58.6 vs. 25.0%; *p* = 0.0156) and during > 24–120 h (93.1% vs. 57.1%; *p* = 0.0020), compared with the FG. Likewise, significantly more vomiting events (269 vs. 136; *p* < 0.0001) were registered in the CG. During HEC, significantly more patients in the CG experienced vomiting during the first 0–24 h (32.3 vs. 9.4%; *p* = 0.0319) compared with the FG. Significantly more vomiting events (241 vs. 99; *p* < 0.0001) were registered in the CG. Laboratory and clinical adverse events were not significantly different between the two groups (*p* > 0.05).

**Conclusions:**

Antiemetic prophylaxis with fosaprepitant and granisetron was well tolerated, safe, and effective in pediatric patients undergoing alloHSCT. However, larger prospective trials are necessary to evaluate these findings.

**Electronic supplementary material:**

The online version of this article (10.1007/s00432-020-03143-8) contains supplementary material, which is available to authorized users.

## Introduction

Children undergoing allogeneic hematopoietic stem cell transplantation (HSCT) receive conditioning chemotherapy for several days containing moderately or highly emetogenic agents with or without total body irradiation (TBI). These regimens are usually accompanied by chemotherapy-induced nausea and vomiting (CINV), representing a severe side effect that substantially impairs the patients’ quality of life (Einhorn et al. 2017; Patel et al. 2017b). Despite recent advances in the development of antiemetics and comprehensive prophylaxis regimens including serotonin-, neurokinin- and dopamine-receptor antagonists, the control rates of CINV in children undergoing HSCT remain unsatisfying (Flank et al. 2017; Kusnierczyk et al. 2002).

The NK_1_ (neurokinin 1) receptor antagonist fosaprepitant (water-soluble prodrug of aprepitant) was recently licensed for pediatric patients between 0.5 and 17 years of age and has shown a good antiemetic efficacy and a good tolerability in pediatric and adult patients undergoing emetogenic chemotherapy (Aapro et al. 2015; Celio et al. 2013; Clemmons et al. 2018; European Medicines Agency (EMA)—Committee for Medicinal Products for Human Use (CHMP) 2018; Radhakrishnan et al. 2018; Saito et al. 2013, 2019; Timaeus et al. 2018; US Food and Drug Administration 2018). Currently, there are no published studies available reporting the use of a fosaprepitant-based antiemetic prophylaxis regimen in pediatric patients undergoing allogeneic HSCT.

The updated pediatric guidelines of the Multinational Association of Supportive Care in Cancer (MASCC) recommend an antiemetic prophylaxis with a 5-HT_3_ (5-hydroxytryptamine 3; serotonin) receptor antagonist (e.g. granisetron) + dexamethasone (+ aprepitant in case of highly emetogenic chemotherapy) for children receiving moderately or highly emetogenic chemotherapy (Dupuis et al. 2017). Based on the favorable experiences, in terms of the safety and efficacy of fosaprepitant, in children undergoing moderately or highly emetogenic chemotherapy, the standard antiemetic prophylaxis regimen at the University Children’s Hospital Tübingen for pediatric patients undergoing allogeneic HSCT was changed from granisetron to a prophylaxis regimen with single-dose intravenous fosaprepitant and granisetron.

In this observational study, the safety and efficacy of single-dose intravenous fosaprepitant in addition to the standard prophylaxis regimen with granisetron in pediatric patients (0.5–17 years of age) undergoing moderately or highly emetogenic conditioning chemotherapy prior to allogeneic HSCT were analyzed and compared to a historical control cohort receiving the standard antiemetic prophylaxis only.

## Patients and methods

### Study background and design

In 2017, the institutional standard antiemetic prophylaxis regimen during allogeneic HSCT consisting of granisetron (moderately and highly emetogenic chemotherapy) was complemented by single-dose, intravenous fosaprepitant directly before starting the first application of a highly or moderately emetogenic agent of the conditioning chemotherapy, due to good experiences with fosaprepitant in pediatric patients undergoing chemotherapy for the treatment of hemato-oncological malignancies.

The primary objective of this analysis was to evaluate the safety and efficacy of the standard antiemetic prophylaxis regimen consisting of granisetron during moderately and highly emetogenic conditioning chemotherapy prior to alloHSCT in comparison to the standard prophylaxis regimen with additional single-dose intravenous fosaprepitant prior to the start of the conditioning chemotherapy in pediatric patients.

A total of 60 consecutive patients who underwent alloHSCT between June 2017 and February 2019 and who met the inclusion criteria were analyzed in the fosaprepitant group (FG). As a control group (CG), 60 consecutive pediatric patients who underwent HSCT between November 2015 and May 2017 and who met the inclusion criteria were analyzed.

The inclusion criteria were undergoing allogeneic HSCT, patient age between 0.5 and < 18 years at the time of HSCT, a conditioning chemotherapy regimen containing moderately or highly emetogenic agents according to the POGO (Pediatric Oncology Group of Ontario) classifications [minimal, stage 1 (< 10% emesis risk); low, stage 2 (10– < 30%); moderate, stage 3 (30–90%); high, stage 4 (> 90%)] (Dupuis et al. 2011), administration of an antiemetic prophylaxis regimen consisting of granisetron only (control group) or granisetron with additional single-dose intravenous fosaprepitant (fosaprepitant group). Antiemetic on-demand medication with dimenhydrinate, metoclopramide or levomepromazine was allowed in both groups.

The exclusion criteria were vomiting or the use of antiemetic drugs or dexamethasone 48 h prior to starting the conditioning chemotherapy, and administration of other NK_1_- or 5-HT_3_-receptor antagonists.

The emetogenic potential of the conditioning before HSCT was determined using the POGO classifications (Dupuis et al. 2011). The emetogenic potential of the total body irradiation used was classified according to international guidelines (McKenzie et al. 2019). The administered agent or irradiation with the highest emetogenic potential defined the overall emetogenic potential (classification as moderately or highly emetogenic conditioning).

The observation period was defined as the time between the first administration of a moderately or highly emetogenic agent of the conditioning until 240 h thereafter. The 240 h were divided into three CINV phases: 0–24 h (acute phase), > 24–120 h (delayed phase), and > 120–240 h. The results of the different analyses were compared to the respective periods.

### Antiemetic prophylaxis

All antiemetic drugs were administered through a central venous catheter or Hickman catheter.

Patients undergoing a moderately or highly emetogenic conditioning chemotherapy received an antiemetic prophylaxis with granisetron (2 × 40 µg per kilogram bodyweight (µg per kg BW) and day; max. 2 × 3 mg per day) during the whole conditioning period; as a slow intravenous (IV) injection within 3 min, starting at least 30 min before starting the conditioning chemotherapy without (control group) or with (fosaprepitant group) fosaprepitant (4.0 mg per kg BW; max. 150 mg; as single-dose IV infusion within 30 min at least 1 h before starting the conditioning chemotherapy).

As on-demand antiemetic medications, dimenhydrinate (3 × 1.0 mg per kg BW per day; max. 3 × 62 mg; as a short IV infusion), metoclopramide (2 × 5–10 mg per day; intravenously), and levomepromazine (0.1 mg per kg BW per day; max. 0.2 mg/kg per day; as 24 h continuous infusion) were allowed.

### Safety and tolerance

During the observation period, adverse events as defined by the United States National Cancer Institute’s Common Terminology Criteria for Adverse Events were documented and analyzed (U.S. NIH -NCI 2010).

Hepatic and renal laboratory markers as well as electrolytes were monitored daily starting on the day of in-patient admission (Baseline) until in-patient discharge. Baseline values as well as minimum/maximum values (Min/Max) during and values at the end (End) of the observation period were analyzed and compared between the study cohorts. Normal blood concentrations of the parameters were defined as alanine aminotransferase (ALT) ≤ 39 U/L (units per liter), aspartate aminotransferase (AST) ≤ 59 U/L, total bilirubin ≤ 1.1 mg/dL (milligram per deciliter), direct bilirubin ≤ 0.3 mg/dL, creatinine ≤ 0.7 mg/dL, urea ≤ 46 mg/dL, potassium 3.4–4.9 mmol/L (millimol per liter), sodium 134 mmol/L—145 mmol/L, and calcium 2.0–2.6 mmol/L. Increases to > 1.5 and > 2.5-fold of the normal limits (ALT, AST, indirect bilirubin, direct bilirubin, creatinine and urea) as well as decreases of sodium (< 134 mmol/L and < 130 mmol/L), potassium (< 3.4 mmol/L and < 3.0 mmol/L), and calcium (< 2.0 mmol/L or and < 1.8 mmol/L) were analyzed and compared between the two groups.

### Efficacy

The efficacy of the antiemetic prophylaxis regimen was determined by the percentage of patients experiencing vomiting, the documented vomiting events, the percentage of patients receiving additional on-demand medication, the number of administered doses of on-demand medication with respect to the administered conditioning regimen (highly or moderately emetogenic conditioning) and the CINV phases (acute 0–24 h, delayed > 24–120 h, and > 120–240 h after the first administration of a moderately or highly emetogenic chemotherapeutic agent).

As primary endpoints, the percentage of patients who did not experience vomiting during the different phases, as well as the percentage of patients with complete control (complete absence of vomiting without the use of on-demand medication), were analyzed and compared.

### Statistical analysis

Fisher’s exact test was used for two-sample tests for equality of proportions and applied to the frequencies of clinical parameters in the two treatment groups (FG and CG). The package rateratio.test of R was used to compare the frequency of the vomiting events and the number of administered doses of on-demand medication between the two study groups.

The Wilcoxon signed-rank test was used to test for differences between the results and the normal range values for the analyzed laboratory parameters.

The Wilcoxon matched-pairs signed-rank test was used for inferential statistical analyses between the baseline values and the maximum/minimum values.

Graphs and statistical tests were created with GraphPad Prism for Windows, version 8 (GraphPad Software Inc., La Jolla, CA, USA), or with R (The R Foundation for Statistical Computing, Institute for Statistics and Mathematics, University of Economics and Business Vienna, Austria). *p* values of *p* < 0.05 (*), *p* < 0.01( **), *p* < 0.001 (***), and *p* < 0.0001 (****) were defined as statistically significant and are illustrated in the bar charts.

## Results

### Patient characteristics

In this analysis, a total of 120 pediatric and adolescent patients (53 females; 44.2%) with a median age of 11.8 years (range 0.7–17.6 years) at the time of HSCT were analyzed. Of these 120 patients, 60 patients each (50.0%) were analyzed in the fosaprepitant group (FG; median age 11.7 years) or in the control group (CG; median age 11.8 years). The patients underwent allogeneic HSCT for the treatment of acute lymphoblastic leukemia (ALL; *n* = 28; 23.3%), acute myeloid leukemia (AML; *n* = 15; 12.5%), beta thalassemia (*n* = 17; 14.2%), Blackfan–Diamond anemia (*n* = 3; 2.5%), myelodysplastic syndromes (MDS; *n* = 9; 7.5%), metachromatic leukodystrophy (*n* = 11; 9.2%), neuroblastoma (*n* = 13; 10.8%), rhabdomyosarcoma (*n* = 3; 2.5%), sickle cell anemia (*n* = 6; 5.0%), or other hemato-oncological malignancies, and non-malignancies (*n* = 15; 12.5%). The patients received stem cells from a mismatched family donor (MMFD; haploidentical HSCT; *n* = 49; 40.8%), a matched unrelated donor (MUD; *n* = 50; 41.7%), or a matched family donor (MFD; *n* = 21; 17.5%). The patients of the CG and the FG were hospitalized, median 32 days (range 15–91 days) and 35 days (range 20–242), respectively, in the transplantation unit until clinical discharge or transfer to a different unit. All patient characteristics including age and age groups, sex, underlying disease or stem cell donor were not significantly different between both study groups (*p* > 0.05) (Table 1).

**Table 1:** Patient characteristics

	Control group	Fosaprepitant group	*p* value
	*N* = 60	*N* = 60	
	*n* (%)	*n* (%)	
Age [years]			
0.5– < 2	2 (3.3)	2 (3.3)	> 0.9999
2–6	18 (30.0)	16 (26.7)	0.8397
7–12	14 (23.3)	21 (35.0)	0.2280
13–17	26 (43.3)	21 (35.0)	0.4546
Sex			
Male	35 (58.3)	32 (53.3)	0.7133
Female	25 (41.7)	28 (46.7)	0.7133
Diagnosis			
Acute lymphoblastic leukemia/relapse	15 (25.0)	13 (21.7)	0.8294
Acute myeloid leukemia/relapse	5 (8.3)	10 (16.7)	0.2693
Beta-thalassemia major	10 (16.7)	7 (11.7)	0.6017
Blackfan–Diamond anemia	2 (3.3)	1 (1.7)	> 0.9999
Myeolodysplastic syndromes	2 (3.3)	7 (11.7)	0.1629
Metachromatic leukodystrophy	2 (3.3)	4 (6.7)	0.6794
Neuroblastoma	7 (11.7)	6 (10.0)	> 0.9999
Rhabdomyosarcoma	3 (5.0)	0 (0.0)	0.2437
Sickle cell anemia	2 (3.3)	4 (6.7)	0.6794
Other	12 (20.0)	8 (13.3)	0.4632
HSCT donor			
Mismatched family donor (haploidentical)	25 (41.7)	24 (40.0)	> 0.9999
Matched unrelated donor	23 (38.3)	27 (45.0)	0.5788
Matched family donor (sibling)	12 (20.0)	9 (15.0)	0.6317
GvHD prophylaxis			
Cyclosporine A	7 (11.7)	4 (6.7)	0.5289
Cyclosporine A + MTX	24 (40.0)	21 (35.0)	0.7063
Mycophenolate mofetil	21 (35.0)	22 (36.7)	> 0.9999
Mycophenolate mofetil + cyclosporine A	2 (3.3)	4 (6.7)	0.6794
Mycophenolate mofetil + cyclosporine A + MTX	2 (3.3)	7 (11.7)	0.1629
Tacrolimus	5 (8.3)	9 (15.0)	0.3945
Anti-thymocyte globulin	58 (96.7)	53 (88.3)	0.1629
Irradiation			
Total body irradiation (total dose: 12 Gy)	5 (8.3)	4 (6.7)	> 0.9999
Total body irradiation (total dose: 4 Gy)	3 (5.0)	1 (1.7)	0.6186

Abbreviations: Gy—Gray; MTX—methotrexate; *N* study cohort size; *n* sample size; *P* value—probability value. No statistically significant difference between the two cohorts (*p* > 0.05)

### Conditioning chemotherapy and irradiation

A brief summary of the administered conditioning chemotherapy and its individual emetogenic potential as defined by the POGO classifications is given in Table 2. Median duration of the conditioning chemotherapy was 8 days (range 7–16 days).

**Table 2:** Emetogenic potential of the conditioning regimen

	Regimen	Agent	Dosage	Control group		Fosaprepitant group	
				MEC [*n*]	HEC [*n*]	MEC [*n*]	HEC [*n*]
1a	Treo/Flu/TT	Treosulfan	3 × 14 g/m^2^	8	4	15	5
		Fludarabine	5 × 30 mg/m^2^				
		Thiotepa	1 × 10 mg/kg				
1b	Treo/Flu	Treosulfan	3 × 14 g/m^2^			3	
		Fludarabine	5 × 30 mg/m^2^				
2a	Bu/Flu/TT	Busulfan	Range 4 × 3.2—4 × 4.0 mg/kg	6	1	4	2
		Fludarabine	4 × 40 mg/m^2^				
		Thiotepa	1 × 10 mg/kg				
2b	Bu/Flu/TT/Cyc	Busulfan	Range 4 × 3.2—4 × 4.0 mg/kg		5		1
		Fludarabine	4 × 40 mg/m^2^				
		Thiotepa	1 × 10 mg/kg				
		Cyclophosphamide	4 × 40 mg/kg or 2 × 60 mg/kg				
2c	Bu/Flu	Busulfan	Range 4 × 3.2—4 × 4.0 mg/kg	1			
		Fludarabine	4 × 40 mg/m^2^				
2d	Bu/Cy/Mel	Busulfan	Range 4 × 3.2—4 × 4.0 mg/kg		1		3
		Cyclophosphamide	2 × 60 mg/kg				
		Melphalan	2 × 70 mg/m^2^				
2e	Bu/Cy	Busulfan	Range 4 × 3.2—4 × 4.0 mg/kg				1
		Cyclophosphamide	2 × 60 mg/kg				
3a	Mel/Flu/TT	Melphalan	2 × 70 mg/m^2^	14	6	6	12
		Fludarabine	4 × 40 mg/m^2^				
		Thiotepa	1 × 10 mg/kg				
3b	Mel/Flu/TT/TLI	Melphalan	2 × 70 mg/m^2^		2		
		Fludarabine	4 × 40 mg/m^2^				
		Thiotepa	1 × 10 mg/kg				
		TLI	7 Gy				
3c	Mel/Flu/TT/Cy	Melphalan	2 × 70 mg/m^2^		1		
		Fludarabine	4 × 40 mg/m^2^				
		Thiotepa	1 × 10 mg/kg				
		Cyclophosphamide	1 × 60 mg/kg				
3d	Bu/Cy/Mel	Busulfan	4 × 4.0 mg/kg				1
		Cyclophosphamide	2 × 60 mg/kg				
		Melphalan	2 × 70 mg/m^2^				
4a	TBI/Eto	TBI	12 Gy		5		4
		Etoposide	1 × 60 mg/kg				
4b	TBI/Eto/TT	TBI	12 Gy				1
		Etoposide	1 × 60 mg/ kg				
		Thiotepa	1 × 10 mg/kg				
5	Flu/TT	Fludarabine	4 × 40 mg/m^2^		2		1
		Thiotepa	1 × 10 mg/kg				
6a	Flu/Cy/TT	Fludarabine	4 × 40 mg/m^2^				1
		Cyclophosphamide	2 × 60 mg/ kg				
		Thiotepa	1 × 10 mg/kg				
		Thiotepa	1 × 10 mg/kg				
6b	Flu/Cy/TBI 4 Gy	Fludarabine	4 × 40 mg/m^2^		3		
		Cyclophosphamide	2 × 60 mg/kg				
		TBI	4 Gy				
6c	Flu/Cy	Fludarabine	4 × 40 mg/m^2^		1		
		Cyclophosphamide	2 × 60 mg/kg				
Total [*n*]				29	31	28	32
(% of cohort)				(48.3)	(51.7)	(46.7)	(53.3)

The table shows the administered conditioning chemotherapy regimens. The overall emetogenic potential of the conditioning chemotherapy was determined by the administered agent with the highest emetogenic potential as determined by the POGO classifications (stages 1–4) (Dupuis et al. 2011). *HEC* highly emetogenic chemotherapy, *MEC* moderately emetogenic chemotherapy, *n* sample size, *P value* probability value. No statistically significant difference between the two cohorts (*p* > 0.05)

Myeloablative conditioning (MAC) with different combinations of chemotherapy (Treo/Flu/TT, Treo/Flu, Bu/Flu/TT, Bu/Flu/TT/Cyc, Bu/Flu, Bu/Cy/Mel, Bu/Cy, Mel/Flu/TT, Mel/Flu/TT/TLI 7 Gy, Mel/Flu/TT/Cy, TBI/Eto, or TBI /Eto/TT) was administered in 112 out of the 120 (93.3%) pediatric patients from which 58 were analyzed in the fosaprepitant group (96.7%) and 54 were analyzed in the control group (90.0%). Reduced-intensity conditioning (RIC) with Flu/Cy/TT, Flu/Cy/TBI 4 Gy, or Flu/Cy was used in 8 of the 120 patients (6.7%) from which 2 were analyzed in the fosaprepitant group (3.3%) and 6 were analyzed in the control group (10.0%).

In total, moderately emetogenic conditioning was administered in 29 (48.3%) and 28 (46.7%) patients of the control and the fosaprepitant group, respectively; highly emetogenic conditioning was administered in 31 (51.7%) and 32 (53.3%) patients of the control and the fosaprepitant group, respectively (*p* > 0.9999). For GvHD prophylaxis, the patients received cyclosporine A (CSA) (*n* = 11; 9.2%), cyclosporine A + methotrexate (*n* = 45; 37.5%), mycophenolate mofetil (*n* = 43; 35.8%), mycophenolate mofetil + cyclosporine A (*n* = 6; 5.0%), mycophenolate mofetil + cyclosporine A + methotrexate (*n* = 9; 7.5%), or tacrolimus (*n* = 9; 7.5%). Anti-thymocyte globulin (ATG) was administered in 111 patients (92.5%).

### Efficacy: moderately and highly emetogenic chemotherapy

Data of all patients were included in the efficacy analysis of the prophylaxis regimen.

In the control group, median 2 (range 1–4) vomiting events per patient occurred within the first 0–24 h, median 2 (range 1–20) during > 24–120 h and median 4 (range 1–15) during > 120–240 h after administration of the first moderately or highly emetogenic agent in the patients experiencing vomiting. In comparison, patients of the fosaprepitant group experienced median 1 (range 1–4) vomiting event per patient during the first 0–24 h (*p* = 0.0005), median 2 (range 1–7) during > 24–120 h (*p* = 0.0039), and median 3 (range 1–14) during > 120–240 h (*p* < 0.0001).

In the fosaprepitant group median 5 doses of dimenhydrinate (range 1–10 doses), 3 doses of metoclopramide (range 2–9 doses), and median 5 days with levomepromazine perfusor (range 3–8 days) were administered per patient. In the control group median 4 doses of dimenhydrinate (range 1–11 doses), 7 doses of metoclopramide (range 2–10 doses), and median 4 days with levomepromazine as continuous perfusor (range 3–5 days) were administered per patient.

**Fig. 1:**
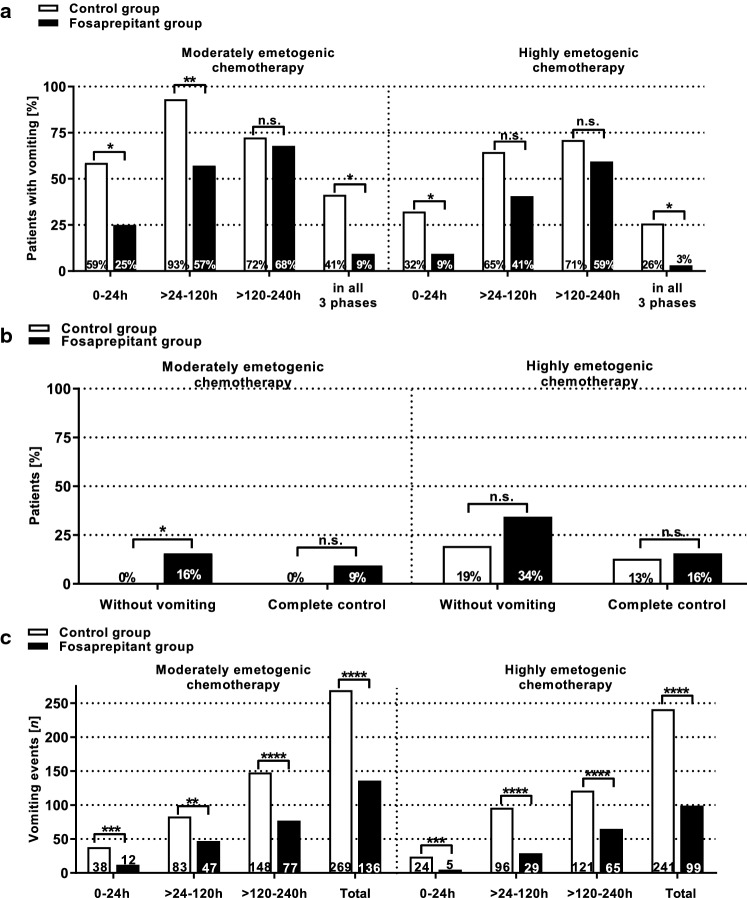
Efficacy of antiemetic prophylaxis. The graph shows the efficacy of the respective anti-emetic prophylaxis regimen during a moderately or highly emetogenic conditioning in patients of the control group (CG) or the fosaprepitant group (FG). In addition to the standard prophylaxis with granisetron, the patients of the FG received single-dose intravenous fosaprepitant before the start of conditioning chemotherapy. **a** In the fosaprepitant group, significantly fewer patients experienced vomiting during the first 0–24 h, > 24 h–120 h, and > 120–240 h after the first administration moderately and highly emetogenic conditioning chemotherapy. **b** The percentage of patients without vomiting during all analyzed time periods was significantly higher in the fosaprepitant group (CG: 0% vs. FG: 15.6%; p = 0.0235) during moderately emetogenic chemotherapy. The number of patients with complete control (complete absence of vomiting without the use of on-demand medication) was not significantly different between both study groups. **c** The number of vomiting events was significantly higher in the control group during all analyzed time periods and during both moderately and highly emetogenic conditioning. Symbols indicate n.s.: not significant; *p< 0.05; **p < 0.01; ***p < 0.001; ****p < 0.0001

During *moderately emetogenic chemotherapy*, percentages of patients experiencing vomiting were significantly higher in the control group during the first 0–24 h (CG: *n* = 17; 58.6% vs. FG: *n* = 7; 25.0%; *p* = 0.0156) and > 24–120 h (CG: *n* = 27; 93.1% vs. FG: *n* = 16; 57.1%; *p* = 0.0020) after the administration of the first moderately emetogenic agent. In addition, significantly more patients (*p* = 0.0148) in the control group (*n* = 12; 41.4%) experienced vomiting in all three analyzed time periods compared to the fosaprepitant group (*n* = 3; 9.4%). Complete absence of vomiting during all three phases occurred in significantly (*p* = 0.0235) more patients of the fosaprepitant group (*n* = 5; 15.6%) compared with the control group (*n* = 0) (Fig. 1).

Significantly more vomiting events occurred in the control group during the first 0–24 h (CG: 38 events vs. FG: 12 events; *p* = 0.0005), during > 24–120 h (CG: 83 events vs. FG: 47 events; *p* = 0.0039), and during > 120–240 h (CG: 148 events vs. FG: 77 events; *p* < 0.0001) (Fig. 1).

**Fig. 2:**
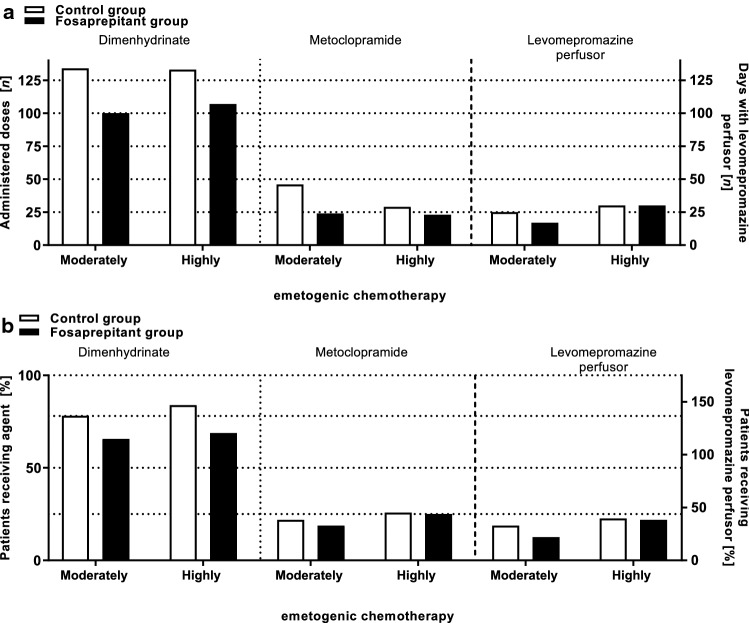
On-demand medication. The graph shows the total number of administered doses (**a**) or the percentage of patients (**b**) receiving on-demand medication with dimenhydrinate, metoclopramide, levomepromazine perfusor during moderately or highly emetogenic chemotherapy in the control or fosaprepitant group. The percentage of patients as well as the number of administered doses of dimenhydrinate, metoclopramide, and levomepromazine was not significantly different (*p* > 0.05) between the two study groups

The percentage of patients receiving dimenhydrinate (CG: 86.2% vs. FG: 75.0%), metoclopramide (CG: 24.1% vs. FG: 21.4%), or levomepromazine continuous perfusor (CG: 20.7% vs. FG: 14.3%) was not significantly different between both cohorts (*p* > 0.05). Administered doses of on-demand medication dimenhydrinate (CG: *n* = 134 vs. FG: *n* = 100; *p* = 0.4068) and metoclopramide (CG: *n* = 46 vs. FG: *n* = 24; *p* = 0.0593) were not significantly higher in the control group compared to the fosaprepitant group. The administered days on which levomepromazine perfusor was used were not significantly different between both study groups (CG: *n* = 25 vs. FG: *n* = 17; *p* > 0.9999) (Fig. 2; Supplementary Table ST1).

During *highly emetogenic chemotherapy*, percentages of patients experiencing vomiting were as well significantly higher in the control group during the first 0–24 h (CG: *n* = 10; 32.3% vs. FG: *n* = 3; 9.4%; *p* = 0.0319) but not > 24–120 h (CG: *n* = 20; 64.5% vs. FG: *n* = 13; 40.6%; *p* = 0.0787) after the administration of the first highly emetogenic agent. Significantly more patients (*p* = 0.0127) in the control group (*n* = 8; 25.8%) experienced vomiting in all three analyzed time periods compared to the fosaprepitant group (*n* = 1; 3.1%). Percentages of complete absence of vomiting (CG: 19.4% vs. FG: 34.4%; *p* = 0.2574) and complete control (complete absence of vomiting without the use of on-demand medication; CG: 12.9% vs. FG: 15.6%; *p* = 0.1132) during all three phases were not significantly different between the two groups (*p* > 0.05) (Fig. 1).

Significantly more vomiting events occurred in the control group during the first 0–24 h (CG: 24 events vs. FG: 5 events; *p* = 0.0004), during > 24–120 h (CG: 96 events vs. FG: 29 events; *p* < 0.0001), and during > 120–240 h (CG: 121 events vs. FG: 65 events; *p* < 0.0001) (Fig. 1).

The percentage of patients receiving dimenhydrinate (CG: 83.9% vs. FG: 68.8%), metoclopramide (CG: 25.8% vs. FG: 25.0%), or levomepromazine continuous perfusor (CG: 22.6% vs. FG: 21.9%) was not significantly different between both cohorts (*p* > 0.05). Administered doses of dimenhydrinate (CG: *n* = 133 vs. FG: *n* = 107; *p* = 0.7473) and metoclopramide (CG: *n* = 29 vs. FG: *n* = 23; *p* = 0.4885) were not significantly higher in the control group compared to the fosaprepitant group. The administered days on which levomepromazine perfusor was used were not significantly different between both study groups (CG: *n* = 30 vs. FG: *n* = 30; *p* > 0.9999) (Fig. 2; Supplementary Table ST1).

### Efficacy: haploidentical HSCT and allogeneic HSCT from a MUD/MFD

Comparing the efficacy parameters with respect to the transplantation type (haploidentical HSCT vs. allogeneic HSCT from a matched unrelated or matched family donor (alloMUD/MFD)), the efficacy of the fosaprepitant prophylaxis regimen was significantly superior to the control group regimen without fosaprepitant.

Although the percentage of patients experiencing vomiting was not significantly different between both haploidentical HSCT subcohorts (*p* > 0.05), the absolute number of vomiting events was significantly lower in the fosaprepitant group compared with the control group during all three time periods (0–24 h: *p* = 0.0074; > 24–120 h: *p* = 0.0004; > 120–240 h: *p* = 0.0017; 0–240 h: *p* < 0.0001). In patients undergoing alloMUD/MFD HSCT, significantly less patients experienced vomiting (0–24 h: *p* = 0.0043; > 24–120 h: *p* = 0.0015; > 120–240 h: *p* = 0.0420; 0–240 h: *p* < 0.0006), and significantly less vomiting events occurred (0–24 h: *p* < 0.0001; > 24–120 h: *p* < 0.0001; > 120–240 h: *p* < 0.0001; 0–240 h: *p* < 0.0001) under fosaprepitant during all three time periods (Supplementary Fig. SF1).

### Safety and tolerance

Discontinuation of the antiemetic prophylaxis was not indicated for any of the patients of the two study groups. None of the patients of the control group died during the hospitalization in the transplant unit. Of the fosaprepitant group, 4 patients (6.7%) died median 105 days (range 48–242 days) after HSCT during the in-patient stay. Reasons for death were severe sepsis (*n* = 1), multi-organ failure (*n* = 1) and graft-versus-host disease °IV (*n* = 2).

Median increases or decreases beyond the normal limits of the analyzed hepatic and renal parameters and electrolytes were detected for ALT (*p* < 0.0001) and direct bilirubin (*p* < 0.001) in both the control and the fosaprepitant group. However, differences of the increases of these two parameters were not significantly different between the two groups (ALT: *p* = 0.4971 | direct bilirubin: *p* = 0.5586). The percentages of increases ≥ 1.5-fold or ≥ 2.5-fold of the upper normal limit of the parameters ALT, AST, indirect bilirubin, creatinine and urea were not significantly different between both cohorts (*p* > 0.05). The increase of transaminases and bilirubin can be most likely ascribed to the administered chemotherapy, especially melphalan and busulfan. No significant difference between both cohorts of clinically relevant decreases of the parameters potassium, calcium, and sodium was detected (*p* > 0.05). Significantly more patients comprised increases of direct bilirubin ≥ 0.45 mg/dL (33.3% vs. 15.0%; *p* = 0.0319) in the control group (*p* = 0.0319) (Supplementary Table ST2).

Clinical adverse events were low in both study groups and included erythema (FG: *n* = 2 vs. CG: *n* = 0), headache (FG: *n* = 1 vs. CG: *n* = 1), sweating (FG: *n* = 2 vs. CG: *n* = 1), itching (FG: *n* = 1 vs. CG: *n* = 0), and constipation (FG: *n* = 2 vs. CG: *n* = 3). No neuropathy symptoms were observed in either group. The occurrence of adverse clinical events was not significantly different in the two study groups (*p* > 0.05).

Fosaprepitant and aprepitant are moderate to weak inhibitors of the CYP3A4, potentially increasing the area under the curve (AUC) of victim drugs such as cyclophosphamide by 1.25-fold up to 5-fold. Concomitant administration of fosaprepitant and other CYP3A4 substrates (e.g. cyclophosphamide) may influence the toxicity and the efficacy of the chemotherapy regimen (Patel et al. 2017a). Blood levels of the relevant concomitantly administered drugs in this analysis (cyclophosphamide, methylprednisolone) were not assessed.

## Discussion

Nausea and vomiting are highly distressing complications in pediatric patients undergoing chemotherapy, increasing the risk for malnutrition, severity of mucositis or graft-versus-host disease and other secondary adverse effects (Flank et al. 2017). Up to 80% of the patients undergoing HSCT experience nausea and vomiting despite the use of a comprehensive prophylaxis regimen, and complete control of CINV is usually achieved in only few pediatric patients (Ballen et al. 2001; Duquette et al. 2011; Flank et al. 2017; Kusnierczyk et al. 2002). The development of new or optimization of existing antiemetic prophylaxis strategies is therefore urgently needed to improve these patients’ quality of life and their treatment outcome. Although the newly available NK_1_-receptor antagonist fosaprepitant has shown a favorable antiemetic efficacy in children undergoing moderately or highly emetogenic chemotherapy (Radhakrishnan et al. 2018; Saito et al. 2019; Shillingburg and Biondo 2014; Timaeus et al. 2018), its use for pediatric patients is not yet proposed by international guidelines (Dupuis et al. 2013, 2017). Study data on fosaprepitant in children undergoing HSCT are currently not available. This is the first report to analyze the efficacy and safety of an antiemetic mono-prophylaxis regimen with granisetron only (control group) in comparison to a regimen consisting of granisetron with additional application of intravenous fosaprepitant in pediatric patients undergoing allogeneic HSCT.

In terms of safety and toxicity of the antiemetic regimen used, a significant difference between the two study cohorts was not detected. None of the patients of both groups was withdrawn secondary to adverse events. New or different adverse reactions to fosaprepitant were not registered.

It was demonstrated that fosaprepitant significantly reduced the percentage of patients experiencing vomiting and the number of vomiting events after both moderately and highly emetogenic conditioning. However, a significantly different use of on-demand medication with dimenhydrinate, metoclopramide, or levomepromazine was not observed between the two groups. To reliably evaluate the effect of fosaprepitant on the experienced nausea, further prospective analyses using a suitable nausea assessment tool are necessary.

Patients undergoing a haploidentical HSCT or an allogeneic HSCT from a MUD or MFD receive a distinctly different conditioning chemotherapy. Patients undergoing haploidentical HSCT (T cell depleted graft) receive ATG and methylprednisolone before starting moderately emetogenic chemotherapy with fludarabine.

In contrast, patients with non-malignant or malignant diseases undergoing allogeneic HSCT with graft from a MUD (alloMUD) receive ATG and methylprednisolone during the administration of emetogenic chemotherapy during the conditioning before HSCT.

Comparing the haploidentical subgroup within the control and the fosaprepitant group, a significant difference of the percentage of patients was not detected, while the number of vomiting events was significantly lower under fosaprepitant. In patients undergoing alloMUD/MFD, the percentage of patients experiencing vomiting and the total number of vomiting events were significantly lower in the fosaprepitant group. The prolonged application of methylprednisolone in patients suffering from non-malignant and malignant diseases and receiving HSCT from a MUD did not comprise a specific antiemetic effect during the three analyzed phases, when compared with patients undergoing haploidentical HSCT and receiving methylprednisolone only during ATG administration before starting moderately or highly emetogenic conditioning chemotherapy.

Similarly, the CINV control rates were considerably higher in a prospective randomized trial analyzing a prophylaxis regimen consisting of fosaprepitant (3 mg/kg BW; single dose), ondansetron (0.3–0.9 mg/kg BW per day; max. 16 mg) and dexamethasone (0.45 mg/kg BW per day), in 163 pediatric patients (median 6 years of age, range 1–12 years) receiving moderately or highly emetogenic chemotherapy only, when compared to the results of this analysis (Radhakrishnan et al. 2018).

In accordance with previously published data on adult patients undergoing HSCT and pediatric patients receiving highly or moderately emetogenic chemotherapy, the results of our analysis demonstrate a clear benefit of the addition of fosaprepitant to the antiemetic prophylaxis regimen during allogeneic HSCT (Grunberg et al. 2011; Radhakrishnan et al. 2018). A comprehensive and effective antiemetic prophylaxis is particularly difficult to accomplish for pediatric patients undergoing allogeneic HSCT. Especially the use of multiple-day chemotherapy regimens with repeated administration of emetogenic agents seems to complicate this task. Larger, prospective trials are urgently needed to significantly improve the antiemetic supportive care in children during HSCT.

## Conclusions

Single-dose fosaprepitant in addition to granisetron was safe and effective as an antiemetic prophylaxis in pediatric patients (0.7–17.6 years of age) undergoing moderately or highly emetogenic conditioning prior to allogeneic HSCT. The antiemetic efficacy was superior to the standard regimen with granisetron only during both moderately and highly emetogenic conditioning. Particularly, additional fosaprepitant significantly reduced the number of vomiting events in the first 240 h after the administration of moderately or highly emetogenic agents. Prospective randomized studies are needed to evaluate these findings.

## Electronic supplementary material

Below is the link to the electronic supplementary material.

Supplementary file1 (EPS 1012 kb)

Supplementary file2 (DOCX 18 kb)

Supplementary file3 (DOCX 22 kb)

## Abbreviations

µg: Microgram
5-HT_3_: 5-Hydroxytryptamine 3; serotonin
ALL: Acute lymphoblastic leukemia
ALT: Alanine aminotransferase
AML: Acute myeloid leukemia
AST: Aspartate aminotransferase
ATG: Anti-thymocyte globulin
BSU: Body surface area
BW: Bodyweight
CG: Control group
CINV: Chemotherapy-induced nausea and vomiting
dL: Deciliter
FG: Fosaprepitant group
Fig.: Figure
HEC: Highly emetogenic chemotherapy
HSCT: Hematopoietic stem cell transplantation
IV: Intravenous
kg: Kilogram
L: Liter
m^2^: Square meter
MASCC: Multinational Association of Supportive Care in Cancer
MDS: Myelodysplastic syndromes
MEC: Moderately emetogenic chemotherapy
MFD: Matched family donor
mg: Milligram
MMFD: Mismatched family donor
mmol: Millimol
MUD: Matched unrelated donor
*N*: Complete cohort size
*n*: Sample size
n.s.: Not significant (*p* > 0.05)
neurokinin-1: NK_1_
*P*: Probability value
POGO: Pediatric Oncology Group of Ontario
TBI: Total body irradiation
U: Units
