# Supplementary material for: Antiemetic prophylaxis with fosaprepitant and granisetron in pediatric patients undergoing allogeneic hematopoietic stem cell transplantation
Source: J Cancer Res Clin Oncol. 2020 Feb 13;146(4):1089–100. doi: 10.1007/s00432-020-03143-8 (PMC7085480; doi:10.1007/s00432-020-03143-8)
Supplement: Supplementary file 2 — Supplementary file2 (DOCX 18 kb) [file 432_2020_3143_MOESM2_ESM.docx]

## Supplementary Table ST1. On-demand medication.

Abbreviations: *N* – study cohort size | *n* – sample size | P-Value – probability value. Fisher’s exact test or rate ratio test in R with packet rateratio.

|  |  |  |  |  |  |
| --- | --- | --- | --- | --- | --- |
|  | **Control group** | | **Fosaprepitant group** | | ***P*-value** |
|  | *N*=60 | | *N*=60 | |  |
|  | *n* (%) | | *n* (%) | |  |
|  |  |  |  |  |  |
|  |  |  |  |  |  |
| **Moderately emetogenic chemotherapy** |  |  |  |  |  |
| Total number of patients | 29 | (48.3) | 28 | (46.7) | >0.9999 |
| **Dimenhydrinate** |  |  |  |  |  |
| Patients receiving agent | 25 | (86.2) | 21 | (75.0) | 0.3313 |
| Administered doses | 134 |  | 100 |  | 0.4068 |
| **Metoclopramide** |  |  |  |  |  |
| Patients receiving agent | 7 | (24.1) | 6 | (21.4) | >0.9999 |
| Administered doses | 46 |  | 24 |  | 0.0593 |
| **Levomepromazine perfusor** |  |  |  |  |  |
| Patients receiving agent | 6 | (20.7) | 4 | (14.3) | 0.7297 |
| Days on which agent was administered | 25 |  | 17 |  | >0.9999 |
|  |  |  |  |  |  |
|  |  |  |  |  |  |
| **Highly emetogenic chemotherapy** |  |  |  |  |  |
| Total number of patients | 31 | (51.7) | 32 | (53.3) | >0.9999 |
| **Dimenhydrinate** |  |  |  |  |  |
| Patients receiving agent | 26 | (83.9) | 22 | (68.8) | 0.2374 |
| Administered doses | 133 |  | 107 |  | 0.7473 |
| **Metoclopramide** |  |  |  |  |  |
| Patients receiving agent | 8 | (25.8) | 8 | (25.0) | >0.9999 |
| Administered doses | 29 |  | 23 |  | 0.4885 |
| **Levomepromazine perfusor** |  |  |  |  |  |
| Patients receiving agent | 7 | (22.6) | 7 | (21.9) | >0.9999 |
| Days on which agent was administered | 30 |  | 30 |  | >0.9999 |
|  |  |  |  |  |  |
