# Supplementary material for: Antiemetic prophylaxis with fosaprepitant and granisetron in pediatric patients undergoing allogeneic hematopoietic stem cell transplantation
Source: J Cancer Res Clin Oncol. 2020 Feb 13;146(4):1089–100. doi: 10.1007/s00432-020-03143-8 (PMC7085480; doi:10.1007/s00432-020-03143-8)
Supplement: Supplementary file 3 — Supplementary file3 (DOCX 22 kb) [file 432_2020_3143_MOESM3_ESM.docx]

## Supplementary Table ST2. Renal and hepatic parameters and electrolytes.

Abbreviations: ALT – alanine aminotransferase | AST – aspartate aminotransferase | n – sample size | mg/dL – milligram per deciliter | mmol/L – millimol per liter | *P*-value – probability value | U/L – Units per liter. Fisher’s exact test.

|  |  |  |  |  |  |
| --- | --- | --- | --- | --- | --- |
| **Laboratory markers** | **Control group** | | **Fosaprepitant group** | | ***P*-value** |
|  | N=60 | | N=60 | |  |
|  | *n* | (%) | *n* | (%) |  |
|  |  |  |  |  |  |
|  |  |  |  |  |  |
| increase ALT \| normal value: ≤39 U/L |  |  |  |  |  |
| ≥1.5 x normal value (≥58.5 U/L) | 16 | (26.7) | 11 | (18.3) | 0.3822 |
| ≥2.5 x normal value (≥97.5 U/L) | 10 | (16.7) | 9 | (15.0) | >0.9999 |
| increase AST \| normal value: ≤59 U/L |  |  |  |  |  |
| ≥1.5 x normal value (≥88.5 U/L) | 10 | (16.7) | 12 | (20.0) | 0.8140 |
| ≥2.5 x normal Value (≥147.5 U/L) | 8 | (13.3) | 4 | (6.7) | 0.3621 |
| increase indirect bilirubin \| normal value: ≤1.1 mg/dL |  |  |  |  |  |
| ≥1.5 x normal value (≥1.65 mg/dL) | 6 | (10.0) | 8 | (13.3) | 0.7772 |
| ≥2.5 x normal value (≥2.75 mg/dL) | 5 | (8.3) | 5 | (8.3) |  |
| increase direct bilirubin \| normal value: ≤0.3 mg/dL |  |  |  |  |  |
| ≥1.5 x normal value (≥0.45 mg/dL) | 20 | (33.3) | 9 | (15.0) | **0.0319** |
| ≥2.5 x normal value (≥0.75 mg/dL) | 9 | (15.0) | 11 | (18.3) | 0.8071 |
| increase creatinine \| normal value: ≤0.7mg/dL |  |  |  |  |  |
| ≥1.5 x normal value (≥1.05 mg/dL) | 0 | (0.0) | 0 | (0.0) | >0.9999 |
| ≥2.5 x normal value (≥1.75 mg/dL) | 0 | (0.0) | 0 | (0.0) | >0.9999 |
| increase urea \| normal value: ≤46mg/dL |  |  |  |  |  |
| ≥1.5 x normal value (≥69 mg/dL) | 0 | (0.0) | 1 | (1.7) | >0.9999 |
| ≥2.5 x normal value (≥115 mg/dL) | 0 | (0.0) | 1 | (1.7) | >0.9999 |
| decrease potassium \| normal value: 3.4 - 4.9 mmol/L |  |  |  |  |  |
| <3.4 mmol/L | 15 | (25.0) | 20 | (33.3) | 0.4220 |
| <3.0 mmol/L | 4 | (6.7) | 0 | (0.0) | 0.1187 |
| decrease calcium \| normal value: 2.0 - 2.6 mmol/L |  |  |  |  |  |
| <2.0 mmol/L | 8 | (13.3) | 10 | (16.7) | 0.7989 |
| <1.8 mmol/L | 0 | (0.0) | 0 | (0.0) | >0.9999 |
| decrease sodium \| normal value: 134 -145 mmol/L |  |  |  |  |  |
| <134 mmol/L | 4 | (6.7) | 7 | (11.7) | 0.5289 |
| <130 mmol/L | 1 | (1.7) | 0 | (0.0) | >0.9999 |
|  |  |  |  |  |  |
